# The Impact of Affective Temperaments on Suicidal Ideation and Behaviors: Results from an Observational Multicentric Study on Patients with Mood Disorders

**DOI:** 10.3390/brainsci13010117

**Published:** 2023-01-09

**Authors:** Mario Luciano, Gaia Sampogna, Bianca Della Rocca, Alessio Simonetti, Pasquale De Fazio, Marco Di Nicola, Giorgio Di Lorenzo, Maria Pepe, Fabio Sambataro, Maria Salvina Signorelli, Alexia Emilia Koukopoulos, Roberto Delle Chiaie, Gabriele Sani, Andrea Fiorillo

**Affiliations:** 1 Department of Psychiatry, University of Campania Luigi Vanvitelli, 80138 Naples, Italy; 2 Department of Neuroscience, Section of Psychiatry, Università Cattolica del Sacro Cuore, 00168 Rome, Italy; 3 Department of Psychiatry, Fondazione Policlinico Universitario Agostino Gemelli IRCCS, 00168 Rome, Italy; 4 Department of Health Sciences, University Magna Graecia of Catanzaro, 88100 Catanzaro, Italy; 5 Department of Systems Medicine, University of Rome Tor Vergata, 00133 Rome, Italy; 6 Department of Neuroscience, University of Padova, 35121 Padua, Italy; 7 Psychiatry Unit, Department of Clinical and Experimental Medicine, University of Catania, 95123 Catania, Italy; 8 Department of Human Neurosciences, Sapienza University, 00161 Rome, Italy

**Keywords:** mood, bipolar, major depression, suicide attempts, suicidal behaviors, suicidality, outcome, temperaments, TEMPS

## Abstract

Suicide ideation and behaviors are major health issues in the field of mental health. Several psychological and psychosocial factors have been taken into account as possible predictors of suicidality. Only recently affective temperaments have been considered as possible factors linked to suicide. This study aims to investigate the relationship between affective temperaments and suicidality, including the lifetime onset of suicide ideation, lifetime presence of suicide attempts and the total number of lifetime suicide attempts. This is a naturalistic multicentric observational study, involving outpatient units of seven University sites in Italy. Patients were administered with the short version of TEMPS-M and the Columbia Suicide Severity Rating Scale. A total of 653 participants were recruited, with a diagnosis of bipolar (55.7%), unipolar (35.8%) and cyclothymic disorder (8.4%). Regression models showed that the presence of lifetime suicide behaviors was increased in patients presenting trait related impulsivity (*p* < 0.0001), poor free-interval functioning (*p* < 0.05), higher number of affective episodes (*p* < 0.01), higher number of hospitalizations (*p* < 0.0001), cyclothymic and irritable affective temperaments (*p* < 0.05 and *p* < 0.05, respectively). Conversely, the presence of hyperthymic affective disposition reduced the likelihood of having suicidal behaviors (*p* < 0.01). Lifetime suicidal ideation was associated with trait-related impulsivity (*p* < 0.001), poor free-interval functioning (*p* < 0.05), higher number of affective episodes (*p* < 0.001) and of hospitalizations (*p* < 0.001). Depressive temperaments increased the likelihood of presenting suicidal ideation (*p* < 0.05), along with irritable temperaments (*p* < 0.01), contrary to hyperthymic affective (*p* < 0.05). Results of the present study confirm that affective disposition has a significant impact on the onset of suicidal ideation and behaviors, and that affective dispositions should be assessed in clinical settings to identify people at risk of suicide. Moreover, a wider clinical evaluation, including different clinical psychopathological dimensions, should be taken into consideration to develop effective preventive interventions.

## 1. Introduction

Suicide is one of the leading causes of death worldwide, accounting for nearly 800.000 deaths every year [1]. It is a complex and multivariate phenomenon, defined as “death caused by self-directed injurious behavior with intent to die being a complex phenomenon” [2]. However, current understanding of suicide as a complex phenomenon do not refer only to completed or attempted suicidal acts, but rather to suicidal ideation, self-injurious, interrupted and aborted (or-self aborted) suicide attempts, and completed suicide [3]. While the global estimates of completed suicides can be assessed, available rates for suicidal ideation, suicide attempts and other self-injurious behaviors could be largely underestimated due to incomplete reporting and to the wide range of available definitions [4]. It has been estimated that rates of suicide attempts are 20 to 40 times higher than completed suicides and that rates of suicidal ideation could be even higher than those of suicide attempts [1,4]. One possible reason explaining the limited availability of data about correlates of suicidality other than suicide attempts is the inadequate understanding on the transition from suicidal ideation to suicide attempts. According to the World Health Organization, the presence of suicide ideation is not associated with an attempted suicide in about two-thirds of patients [5]; moreover, the possible mechanisms behind the transition from suicide ideation to suicide attempts have been investigated only in a few studies. Thus, suicidality has been mainly explored in terms of suicidal behaviors, since they can be more easily detected, and the result is that little is known about predictors of suicidal ideation and on the consequences of multiple suicide attempts in terms of patients’ illness severity, psychosocial functioning, and other psychopathological outcomes.

Moreover, it has to be said that rates of suicidality as a whole will likely increase during and after the COVID-19 pandemic, which affects mental and physical health, economy, and social life, increasing patients’ social isolation and feelings of loneliness [6].

Suicide is considered a major health issue [7]. There is a growing interest in the identification of suicide predictors, especially those that can be targeted with appropriate psychosocial health interventions, and its clinical correlates, including the association with psychiatric diagnoses, personality, and temperamental traits [8,9].

Psychiatric disorders are an important contributing factor to suicide [10], and, among all, major depressive disorder (MDD) and bipolar disorders (BDs) are associated with the highest suicide risk [11,12], being 5–10 times higher than the general population [13,14] and being associated with a greater lethality (from 10 to 30 times higher than the general population) [15]. Of concern, the risk for suicidal behaviors and ideation is higher early in the course of mood disorders (MDs) and among young people. Therefore, efforts to identify reliable and readily accessible clinical dimensions associated with suicide is of crucial importance both at clinical and research levels. It has been suggested that, although suicide is more frequently associated with specific psychiatric diagnoses, it is not the diagnosis per se that can be considered a clinical predictor of suicide attempt or ideation, but rather several psychopathological dimensions, including mental pain, emotional dysregulation, impulsivity, low self-esteem, or hopelessness, which could potentially mediate the association between affective disorders and higher rates of suicide ideation and attempts [16,17,18,19,20,21,22].

Personality and temperamental traits have also been considered possible predictors of suicidality [23]. Recently, affective temperaments (ATs) have been proposed as possible risk factors for suicidality [24]. Traditionally, ATs represent the “temporally stable biological core of personality” influencing several aspects of an individual’s life (activity levels, rhythms, mood, and related cognitions), while personality, a broader phenotype, refers to acquired characterological determinants [25]. ATs are considered stable traits related to the expression of emotion, resulting in a variability of reactivity, energy levels and self-regulation [26]. Based on the initial description provided by Kraepelin, the current conceptualization of ATs includes: (1) hyperthymic temperaments, characterized by cheerful, energetic and reckless traits; (2) anxious disposition, defined by a significant attitude to worrying; (3) depressive temperaments, whose key characteristic is the tendency to mental suffering, and to complying with social role and rules and self-denying devotion to others; (4) cyclothymic disposition, describing individuals with rapid mood and energy shifts; (5) irritable disposition, which is associated with an unpredictable temperament with frequent impulsive thinking and behaviors [26,27,28]. Moreover, the clinical manifestations of ATs are mainly related to emotional reactivity to internal and external stimuli, cognitive styles, vitality, cognitive skills, and individual responses to stressful situations [29]. Of note, behavioral and cognitive aspects of affective dispositions are not listed among operational criteria for diagnosing MDs in the Diagnostic and Statistical Manual for Mental Disorders, fifth edition (DSM-5) and in the diagnostic guidelines of the International Classification of Diseases, 11th revision (ICD-11). This reflects difficulties of current diagnostic manuals to catch the heterogeneity of clinical presentations of MDs and the complex interaction among symptoms and their underlying cognitive and emotional processes [30,31]. Moreover, available research shows that the five affective dispositions cannot be conceptualized as categorical entities, but rather as psychological dimensions that lie on a continuum from adaptive behaviors to different degrees of psychopathological manifestations.

Available research, carried out on clinical and non-clinical samples, highlights that affective dispositions could be considered the subclinical (trait-related) manifestations and, in some cases, precursors of mood disorders [32]. However, affective dispositions could significantly influence the clinical presentation of mood disorders, including polarity, symptom characterization and long-term outcome.

The existing relationship among ATs and suicide ideation and behaviors has been assessed in the general population [33,34,35] and in clinical populations, including patients with psychosis, anxiety and affective disorders [36,37,38]. With some exception, there is an agreement among studies that hyperthymic temperament constitutes a protective factor against suicide [20,39], whereas cyclothymic, irritable, depressive and anxious temperament are reported more frequently by individuals with a history of suicide attempts [40,41]. Cyclothymic and irritable temperaments are highly connected with both aggressiveness [42] and impulsivity [23], often associated with suicidality. Mechanisms explaining the associations between ATs and suicidality still lack a clear explanation. Some affective dispositions, including cyclothymic and irritable, are often associated with sleep disturbances, sensitivity to separation, and antisocial aspects, suggesting a possible relationship among these two dispositions with high levels of impulsiveness, which is considered a risk factor for suicide attempts [43]. Another possible explanation of this association lies in some biological commonalities between ATs and suicidality. In fact, the expression of the “S” allele of the serotonin transporter (5-HTTLPR) is associated with a higher incidence of suicide attempts [28] and of specific ATs, especially the cyclothymic one [28].

However, available studies have included small sample sizes, or have been carried out with acute patients, in which the reporting of temperamental characteristic could have been biased by the presence of active symptoms. Moreover, the majority of studies did not take into account the geographical distribution of samples, with one or at least two recruiting centers, providing results not directly generalizable. Moreover, the majority of sample took into account only one aspect of suicidality (i.e., the presence of suicide attempts) and did not assessed suicidality with different means, including, as an example, the onset of suicidal ideation or the total number of suicide attempts, which are per se considered a clinical proxy of a more severe outcome of the disorder.

Therefore, we decided to carry out a multicentric observational study, in inpatients suffering from affective disorders (both unipolar than bipolar affective disorders), with the aim to investigate the relationship between ATs and suicidality, including the lifetime onset of suicide ideation, presence of suicide attempts in patients’ anamnesis and the total number of lifetime suicide attempts.

We hypothesized that affective temperaments could have a role in influencing suicidal behaviors and ideation, in the sense that hyperthymic disposition could be associated with a reduced onset of suicidal ideation and behaviors, while cyclothymic, irritable, anxious, and depressive ones to a worse suicide profile, with higher number of lifetime suicide attempts and increased likelihood to have suicidal ideation lifetime.

## 2. Methods

This is a naturalistic multicentric observational study, involving university centers of Campania “L. Vanvitelli”, Catania, Magna Graecia of Catanzaro, Cattolica del Sacro Cuore of Rome, Padova, Sapienza University of Rome and Tor Vergata of Rome. In the period between November 2020 and January 2021, patients in the mood disorder units of participating centers were consecutively enrolled if they: (1) had a main diagnosis of type-I or type-II BD, MDD or cyclothymic disorder, according to DSM-5 criteria; (2) were aged 18–65 years; (3) were in a stable phase of their illness, as defined by a total score less than 9 at the Hamilton Rating scale for Depression [44] and a score ≤ 11 at the Young Mania Rating Scale [45]; (4) were receiving medications for their major depressive disorder or bipolar disorder according to the most recent NICE guidelines [46,47]. Patients were excluded if they: (1) were unable to provide consent; (2) presented a neurological disorder or substance abuse. The study was approved by the Local Research Ethic Committee of the coordinator center (Protocol number ID 5016) and was carried out according to the Declaration of Helsinki.

### 2.1. Procedures

Patients’ socio-demographic and clinical characteristics were recorded with an ad hoc schedule. Previous mood episodes have been defined according to DSM-5 criteria.

Suicidality was assessed using the Columbia Suicide Severity Rating Scale (C-SSRS), which rates suicidal ideation and attempts [48]. We used the clinician-administered version of the C-SSRS (screening version) to assess lifetime ratings for suicidal ideation and behaviors. Each individual’s suicidal ideation is rated on a scale from 1 (wish to be dead) to 5 (active suicidal ideation with a specific plan and intent). According to suggested scoring for suicidal ideation, all patients were rated as positive for suicide ideation if they responded positively at one of the five items assessing suicidal ideation. Individual previous suicidal attempts are rated on a scale from 1 (actual attempt, defined as “a potentially self-injurious act committed with at least some wish to die, as a result of act”) to 4 (preparatory acts or behavior, intended as “Acts or preparation towards imminently making a suicide attempt”). All patients were rated as positive for suicide attempts if they responded positively to one of the four items assessing suicidal behaviors. The C-SSRs have been used in several trials and has been extensively validated in different populations, both with clinical and non-clinical samples at higher risk of suicide. Validation studies have reported a sensitivity of 95.0% and a specificity of 95% when the questionnaire has been used in outpatients with severe mental disorders [49]. The C-SSRS is recommended by the US Food and Drug Administration and has been adopted by the Centers for Disease Control and Prevention to define and stratify suicidal ideation and behaviors [50].

The short version of the Munster Temperament Evaluation of the Memphis, Pisa, Paris and San Diego (b-TEMPS-M) was adopted to evaluate ATs. B-TEMPS-M is a 35-item questionnaire, whose factor analyses led to the identification of the five ATs, according to Akiskal classification [51]. Cronbach’s alpha coefficients for the b-TEMPS-M subscales were included within 0.808 and 0.898, while Kaiser Meyer-Olkin (KMO) was reported to be 0.914, corresponding to the recommended value of at least 0.6; Bartlett’s Test of Sphericity was statistically significant (*p* < 0.0001), supporting the factorability of the correlation matrix and confirming the five-factor model of the questionnaire.

Global functioning during symptom-free intervals was assessed with the Global Assessment of Functioning GAF) [52], a 100-point rating scale assessing social, occupational, and psychological functioning of adults. Patients were coded as impaired according to a total score higher than 61. The intraclass correlation coefficients for GAF has been reported to be 0.81 (95% CI ranging from 0.65 to 0.95) when administered to outpatients with severe mental disorders [53].

The Barratt Impulsiveness Scale (BIS-11) was adopted to assess impulsiveness. BIS-11 items are rated on a Likert scale (1 = rarely, 4 = almost always/always). BIS-11 total score ranges from 30 to 120. BIS-11 total score has revealed a good internal consistency (Cronbach’s α = 0.83) and reliability (Spearman’s Rho = 0.83) across validation studies. A total score ≥ 72 is indicative of the presence of trait-related impulsiveness in outpatients with severe mental disorders [54].

### 2.2. Statistical Analyses

Descriptive statistics were calculated for socio-demographic and clinical characteristics, as well as for scores of relevant assessment instruments. Data were presented as means (M) and standard deviations (SD), or as frequencies and percentages (%), as appropriate. The Kolmogorov–Smirnov test was used to check the normality of distribution of our sample.

Sample was divided according to the C-SSRS positivity for suicidal attempts in patients positive for lifetime suicidal behaviors (SBG) and those negative for lifetime suicidal behaviors (NSBG). Differences among groups were calculated with the t-Student test of χ^2^ as appropriate. Moreover, the χ^2^ test was used to assess the association among ATs, BIS-11 total score and GAF score and the presence of lifetime suicide behaviors, lifetime suicidal ideation and lifetime number of suicidal attempts. With regard to BIS-11 total score, according to Stanford et al. [54], the sample was divided into those with trait-related impulsivity (BIS-11 ≥ 72, coded as 1) and those without trait-related impulsivity (BIS-11 < 72, coded as 0).

Three different logistic regression analyses were performed to test the association among the presence of lifetime suicide behaviors, lifetime suicidal ideation and lifetime number of suicidal attempts (dependent variables) and all other socio-demographic and clinical variables, selected among those who were statistically significant at univariate analyses and others identified by the relevant literature (including diagnosis, number of affective episodes, psychiatric hospitalizations and number of involuntary hospitalizations, seasonality, presence of psychotic symptoms during affective episodes and affective temperaments).

## 3. Results

### 3.1. Descriptive Analysis of Total Sample

A total of 653 participants were recruited, consisting of 55.7% of patients affected by BDS, 35.8% by MDD and 8.4% by cyclothymic disorder (CYC) and with a mean age of onset of the disorder of 31.3 ± 13.1 years. Most of the sample included female participants (58.2%), with a mean age of 46.9 ± 14.1 years. Thirty-nine percent of patients lived with a partner, and 54.7% were employed. A total of 17.9% of recruited patients reported at least one suicide attempt. Patients reported 4.5 ± 5.0 mean previous affective episodes. Patients reported to have a mean of 2.4 ± 2.5 number of previous voluntary hospitalizations.

### 3.2. Differences among Patients with and without Suicide Attempts

In the present sample, 125 patients out of 653 reported at least one suicide attempt during their lifetime. Patients in the suicidal group (SG) were more often females (62.4%), mean age was 44.8 ± 13.9, and educational level was 13.4 ± 3.5. Patients in the non-suicidal group (NSG) were mostly females (57.5%), with a mean age of 31.8 ± 13 years. (Table 1).

A significantly higher proportion of SG patients reported the presence of trait-related impulsiveness (81.6% in the SG vs. 48% in the NSG group, *p* < 0.0001). Seasonality was significantly more present among the SG group (39.2% vs. 30.8% in the NSBG, *p* < 0.001). Furthermore, patients in the SG experienced psychotic symptoms more frequently compared to non-suicidal group (39.2% vs. 27.4%, *p* < 0.05). In addition, global functioning during stable phases of the disorder was more frequently reported as impaired in suicidal patients (73.7% vs. 58%, *p* < 0.0001). Moreover, SG patients reported a higher mean number of affective episodes (5.3 ± 7.5 vs. 3.2 ± 3.3, *p* < 0.0001), higher mean number of hospitalizations (2.8 ± 3.6 vs. 2.2 ± 1.6, *p* < 0.001) and of involuntary hospitalizations (0.2 ± 0.7 vs. 0.2 ± 0.7, *p* < 0.05).

Moreover, patients in the SG group presented more frequently impaired psychosocial functioning (12.6% of cases vs. 23.1% in the NSG, *p* < 0.001) and trait impulsiveness during stable phases of the disorder (28.7% vs. 7.7% in the NSG, *p* < 0.0001).

### 3.3. Suicidality, Affective Temperaments and Other Clinical Variables

The majority of patients showed a depressive temperamental disposition (28.9%), followed by cyclothymic (23.9%), hyperthymic (23.4%) anxious (14.4%) and irritable (8.1%).

**Figure 1:**
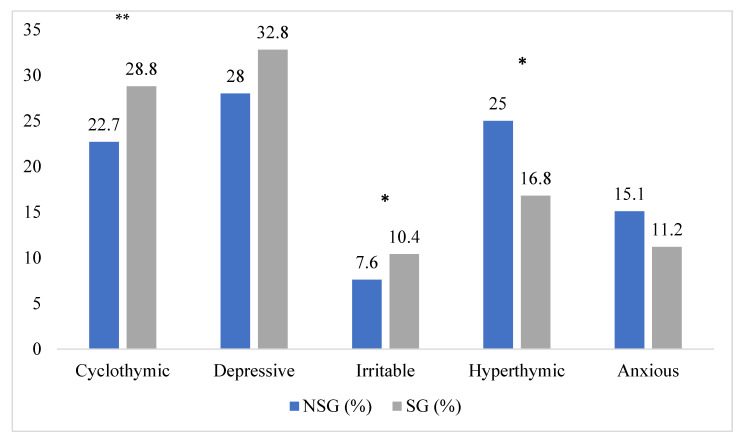
Distribution of suicide attempts lifetime among affective temperaments. * *p* < 0.01; ** *p* < 0.001; NSG: non suicidal group; SG: suicidal group.

Cyclothymic temperamental disposition was more frequently associated with suicidal behaviors (28.8% vs. 22.7%, *p* < 0.001), along with irritable temperament (10.4% vs. 7.6%, *p* < 0.01). Conversely, the number of patients with suicidal behaviors was significantly lower within the hyperthymic group (25% vs. 16.8%, *p* < 0.01). (Figure 1).

**Figure 2:**
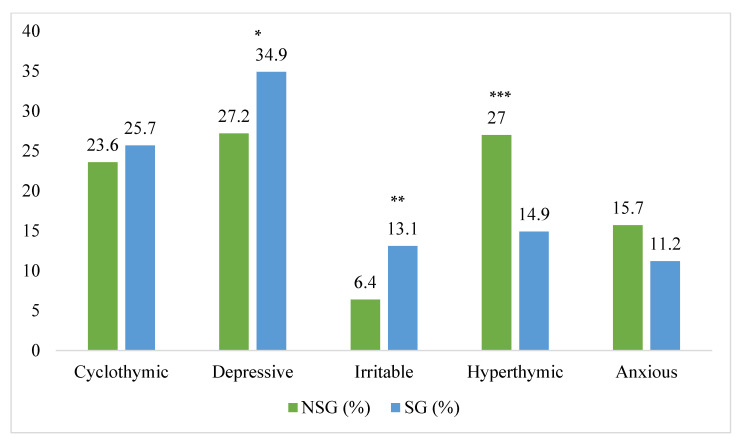
Distribution of suicide ideation lifetime among affective temperaments. * *p* < 0.05; ** *p*< 0.01; *** *p* < 0.001; NSG: non suicidal group; SG: suicidal group.

Hyperthymic temperament was significantly less frequently associated with suicide ideation (14.9% in those who presented lifetime suicide ideation vs. 27% who did not report lifetime suicide ideation, *p* > 0.001). Suicide ideation was more frequently present in depressive (34.9% vs. 27.2%, *p* < 0.05) and irritable patients (13.1% vs. 6.4%, *p* < 0.01) (Figure 2).

Moreover, patients with lifetime suicide ideation more frequently presented trait-related impulsivity (36.5% vs. 15.1%, *p* < 0.0001) and impaired psychosocial functioning (23.1% vs. 12.6%, *p* < 0.0001) compared to the rest of the sample.

**Figure 3:**
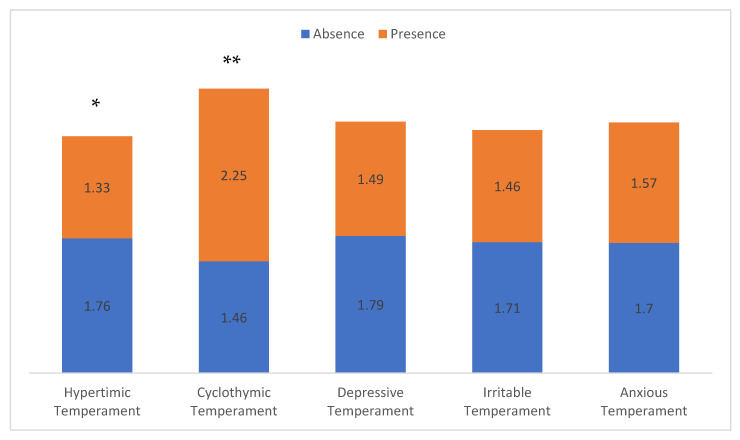
Number of suicide attempts according to dominant affective temperament. * *p* < 0.05, ** *p* < 0.001.

In addition, cyclothymic temperament reported a significantly higher number of lifetime suicide attempts (2.25 vs. 1.46, *p* < 0.001). Conversely, hyperthymic patients showed a lower number of lifetime suicide attempts (1.75 vs. 1.33, *p* < 0.05) (Figure 3).

Patients with trait-related impulsiveness presented a higher number of suicide attempts (1.29 vs. 0.67 in the rest of the sample, *p* < 0.0001) and a more frequently impaired psychosocial functioning (1.23 vs. 0.56 in the rest of the sample; *p* < 0.001).

### 3.4. Logistic Regression Analysis

Regression models showed that the presence of lifetime suicide behaviors was increased in patients presenting: (1) trait related impulsivity (OR: 3.89; 95% CI: 2.3 to 6.58; *p* < 0.0001); (2) poor free-interval functioning (OR: 1.72; 95% CI: 1.05 to 2.81; *p* < 0.05); (3) number of affective episodes (OR: 1.08; 95% CI: 1.03 to 1.13; *p* < 0.01); (4) number of psychiatric hospitalizations (OR: 4.45; 95% CI: 2.67 to 7.41; *p* < 0.0001); (5) cyclothymic and irritable affective temperament (OR: 1.08; 95% CI: 0.5 to 2.32; *p* < 0.05 and OR: 1.19; 95% CI: 0.47 to 3.02; *p* < 0.05, respectively). Conversely, the presence of hyperthymic affective disposition reduced the likelihood of having suicidal behaviors (OR: 0.8; 95% CI: 0.35 to 1.81; *p* < 0.01) (Table 2).

Moreover, lifetime suicidal ideation was associated with: (1) trait-related impulsivity (OR: 2.67; 95% CI: 1.74 to 4.08; *p* < 0.001), (2) poor free-interval functioning (OR: 1.7; 95% CI: 1.11 to 2.6; *p* < 0.05), (3) number of affective episodes (OR: 1.08; 95% CI: 1.03 to 1.13; *p* < 0.001), and (4) psychiatric hospitalizations (OR: 2.7; 95% CI: 1.71 to 4.26; *p* < 0.001). Moreover, depressive temperaments increased the likelihood of present suicidal ideation (OR: 1.67; 95% CI: 0.89 to 3.16; *p* < 0.05), along with irritable temperaments (OR: 2.15; 95% CI: 0.96 to 4.8; *p* < 0.01). On the contrary, hyperthymic affective disposition reduced the probability to develop lifetime suicidal ideation (OR: 0.7; 95% CI: 0.34 to 1.43; *p* < 0.05) (Table 2).

With respect to total number of suicide attempts, regression analyses reported that the number of suicidal attempts was positively associated to: (1) trait-related impulsivity (OR: 0.34, 95% CI: 0.2 to 0.47, *p* < 0.001), (2) poor free-interval functioning (OR: 0.18, 95% CI: 0.05 to 0.31, *p* < 0.01), (3) number of affective episodes (OR: 0.02, 95% CI: 0.01 to 0.03), and (4) psychiatric hospitalizations (OR: 0,49, 95% CI: 0.34 to 0.65, *p* < 0.0001). In addition, patients with cyclothymic temperamental disposition were more likely to have a higher number of suicide attempts (OR: 0.14, 95% CI: −0.17 to 0.35, *p* < 0.05). Conversely, hyperthymic temperament was associated with a lower total number of suicide attempts (OR: −0.14, 95% CI: −0.35 to 0.37, *p* < 0.01) (Table 3).

## 4. Discussion

To our knowledge, this is one of the few papers assessing the relationship existing among affective dispositions and different aspects of suicidality assessed with a structured and validated questionnaire in patients with MDs. We decided to focus on clinical populations of patients with MDs, without active symptoms and currently on medication according to the relevant NICE guidelines. The choice to focus on clinical population is due to the fact that studies on the association between affective temperaments and suicide behaviors and ideation in the general population are already available [35,55], and that individuals with MDs are usually considered at higher risk of developing suicide ideation and behaviors, compared to the general population and to individuals with mental disorders other than MDs. Moreover, we decided to recruit only stable patients, since report of ATs can be biased by the presence of acute symptoms. This choice represents one strength of the present paper, since the majority of available studies, with some exceptions, have been carried out in acutely ill patients. Although ATs are considered stable traits of personality, their assessment relies mainly on self-reported questionnaires, which can be biased by the presence of severe affective symptoms. Therefore, its assessment during stable phases of affective disorders is crucial in assessing the real impact of affective disposition on different aspects of suicidality. Moreover, the multicentric design of the study, the large sample size and the naturalistic setting, make results of the present study generalizable to patients with affective disorders.

With respect to the first aim of the present paper (i.e., are affective temperaments associated with suicidality?), findings from the present study provide further evidence that hyperthymic temperament is strongly linked to a reduced risk of suicidality as a whole. These findings are in line with previous reports, in which hyperthymic temperament has been considered a protective factor for suicidality. Individuals with hyperthymic temperaments usually present better drive, greater energy, more ambition, as well as better coping and decrease the risk of suicidal behavior. These characteristics could explain the protective role of this AT on suicide risk. Moreover, it has been reported that patients with hyperthymic temperament, both in clinical that non-clinical samples, presents greater confidence, sociability, and an optimistic view toward life [56,57], which are considered protective factors for suicidality. Low ratings of hyperthymic disposition are associated with increased hopelessness and negative attitudes toward life [58].

On the contrary, in the present study cyclothymic and irritable temperaments were associated with different aspects of suicidality, in line with previous reports [16,22,59]. Mechanisms by which these two affective temperaments influence suicidal behaviors, in terms of lifetime number of suicide attempts and presence of previous attempts, include the emotional instability, marked mood reactivity to lifetime stressors, rapid mood fluctuations [4]. According to this, cyclothymic and irritable temperaments, which are often characterized by abrupt shifts in behaviors and mood, may represent a specific marker for suicidality in patients with both bipolar than unipolar affective disorders [22]. One possible mediator of this association is emotional dysregulation and impulsivity. In fact, it has been demonstrated that patients with suicidal ideation, showed a reduced performance in emotion regulation tasks, deficits in decision-making control processes and impaired emotional regulation [60,61], which results in increased in a higher trait- and state-related impulsivity. This hypothesis is confirmed by findings of the present study since higher BIS-11 impulsivity total score were associated with all dimensions of suicidality. The presence of a strong association among impulsivity and suicidality has been reported by previous studies [62]. Zhang et al. [63] highlighted that impulsiveness could be a possible moderator of suicidality. In fact, impulsiveness can be considered a predictive factor for the onset of depressive symptoms, which are directly correlated to suicidal ideation.

Moreover, according to study results, higher levels of impulsivity could represent a significant contributing factor to mood instability, which negatively influences patients’ skills to effectively manage stressful situations. Accordingly, the presence of impulsivity in patients with affective disorders account for a distinct phenotype, characterized by rapid, unplanned reactions, failure in understanding negative consequences of those reactions, attentional bias and cognitive rigidity and reduced coping strategies [64].

Other characteristics emerged as strongly associated with suicidality. Among them, the presence of and impaired psychosocial functioning during euthymic phases, the total number of affective episodes and a higher number of previous psychiatric hospitalizations. The relationship between poor psychosocial functioning and the severity of illness has been poorly studied in MDs. In fact, the long-term effect of a reduced functioning in daily life have been extensively studied mainly in psychotic disorders, while poor evidence exists with respect to patient with MDs [65]. Wang et al. [64] reported that individuals with impaired psychosocial functioning during stable phases presented more physical symptoms, more residual symptoms of depression, and less satisfaction with quality of life. It may me that patients with reduced psychosocial functioning, especially during the stable phases of the disorder, have reduced skills to cope with stressful life events, impaired autonomy, cognitive deficits [65,66,67] and therefore could have a worse long-term outcome, which include the onset of suicidal ideation and the presence of suicidal attempts.

These results further support the notion that psychosocial functioning should be assessed in clinical settings not only when the presence of symptoms could have a significant impact on patients’ autonomy, but also after an acute episode, since impairment in free-interval functioning could impact suicidality, relapses, and hospitalizations. Therefore, ad hoc non-pharmacological interventions should be offered to patients with affective disorders, with the aim of promoting the process of recovery.

Interestingly, it has been noted that several psychological, psychosocial, and temperamental factors were strongly associated with suicidality more than diagnoses themselves, suggesting that the diagnostic categories are still not able to describe the complexity underlying MDs [68,69]. This result supports the evidence that MDs belong to a broad affective spectrum, where ATs, personality characteristics and other psychopathological dimensions delineate multiple clinical phenotypes [70]. Some authors [71,72] have criticized the existing dichotomy between unipolar and bipolar MDs, suggesting a broader conceptualization of mood disorders, echoing the manic-depressive continuum theorized by Kraepelin [73,74]. This approach might guide clinicians toward a better clinical characterization and personalized treatment plan [75,76], suggesting the need for a change of paradigms in mental health, which includes a transdiagnostic approach to mental disorders, and seems more useful for clinical practice than the categorical classification [77,78,79].

Results of the present paper should be considered considering several limitations. First, the cross-sectional design precludes any consideration regarding cause–effect relationships. Longitudinal studies are warranted in order to ascertain the potential impact of affective temperaments on suicidality. Second, there is a lack of a control group made by patients affected by non-affective mental disorders; therefore, we could not clarify if results of the present study are disease-specific or can be applicable to other non-affective mental disorders. Another possible limitation is the fact that suicidal ideation and behaviors have been retrospectively assessed. However, compared to the majority of available studies, we adopted a structured and validated questionnaire to assess suicidality, the Columbia Suicide Severity Rating Scale, in order to reduce the recall bias. Moreover, data on patients who were screened and not included in the study because they did not meet inclusion criteria were not collected. Lastly, we did not consider patients’ physical and psychiatric comorbidities since this was not the primary outcome of this manuscript. However, we plan to analyze and publish data on physical and psychiatric comorbidities of our sample in subsequent papers.

## 5. Conclusions

Findings of the present study confirm the presence of a strong association between several aspects of suicidality, affective temperaments and other psychopathological dimensions in patients suffering from affective disorders. While available literature has been mainly focused on the relationship between affective dispositions and previous suicide attempts, our study has included the assessment of different aspects of suicidality (i.e., suicidal ideation, previous number of suicidal ideation and presence of suicide attempts lifetime). According to the study results, we can conclude that individuals with MDs presenting a cyclothymic or irritable disposition are at higher risk for suicidality. Conversely, the hyperthymic disposition is protective against the development of suicidal ideation and behaviors. Interestingly, affective temperaments influence suicidality more strongly than the diagnoses themselves, further supporting the need to develop a dimensional and transdiagnostic approach to mental disorders, as opposed to the categorical one. Moreover, we also found that other clinical features, commonly associated with MDs, such as trait-related impulsivity and impaired psychosocial functioning, could greatly contribute to the increased risk for suicidality in patients with specific affective temperaments. Therefore, it is of utmost importance to routinely assess affective dispositions in clinical settings to identify people at higher risk of suicide. Moreover, the development of programs for suicide prevention should seriously consider the presence of specific affective temperaments, although it would be reductive to build a “suicide attempter profile” only on the basis of temperaments. A wider clinical evaluation, including different clinical aspects such as current affective episodes and severity of the disease, psychosocial functioning, and the use of coping strategies in stressful situations, should be taken into consideration to develop effective interventions. Moreover, ad hoc interventions should be developed, also taking advantage of new technologies [80,81] and social media, which can booster the scalability of such interventions.

## Figures and Tables

**Table 1:** Differences in sociodemographic and clinical characteristics among patients with and without suicide attempts.

	NSG (N = 529)	SG (N = 125)
Age (M ± DS)	47.6 (14)	44.8 (13.9)
Gender, Male % (N)	42.5 (225)	37.6 (47)
Years of education (M ± DS)	13.2 (3.7)	13.4 (3.5)
Age at onset (M ± DS)	31.8 (13)	29.4 (13)
Marital status, % (N) Single Married Widowed Divorced	40.1 (212) 39.7 (210) 3.4 (18) 16.6 (88)	44.8 (56) 35.2 (44) 1.6 (2) 18.4 (23)
Occupation, yes. % (N)	55.3 (265)	53.1 (93)
Diagnosis, % (N) Bipolar disorder Major depressive disorder Cyclothymic disorder	53.7 (284) 37.4 (198) 8.7 (46)	64 (80) 28.8 (36) 7.2 (9)
Family history of psychiatric diseases, yes. % (N)	55.8 (295)	56 (70)
Impulsivity, yes, % (N)	48 (254)	81.6 (102) ****
Seasonality, yes, % (N)	30.8 (163)	39.2 (49) ***
Presence of psychotic symptoms during acute phases, yes, % (N)	27.4 (145)	39.2 (49) *
Insight of illness, % (N) Absent Partial Present	6 (32) 38 (201) 56 (296)	5.6 (7) 39.2 (49) 55.2 (69)
Adherence to treatment Absent Partial Present	5.1 (27) 26.3 (139) 68.6 (363)	8 (10) * 28.8 (36) 63.2 (79)
Functioning during disease-free interval, % (N) Impaired Non-impaired	58 (278) 42 (201)	73.7 (129) **** 26.3 (46)
Hospitalizations, yes, % (N)	171 (32.3)	65.6 (82) ****
Involuntary hospitalizations, yes, % (N)	11.5 (61)	17.6 (22)
Previous mood episodes (M ± DS)	3.2 (3.3)	5.3 (7.5) ****
Previous voluntary hospitalizations (M ± DS)	2.2 (1.6)	2.8 (3.6) **
Number of involuntary hospitalizations (M ± DS)	0.2 (0.5)	0.2 (0.7) *

* *p* < 0.05 ** *p* < 0.01; *** *p* < 0.001; **** *p* < 0.000. NSG: non suicidal group; SG: suicidal group.

**Table 2:** Logistic regression analyses.

	Presence of Suicide Attempts Lifetime			Presence of Suicide Ideation Lifetime		
	OR	95% CI			95% CI	
		Lower Bound	Upper Bound	OR	Lower Bound	Upper Bound
Diagnosis of bipolar disorder	0.680	0.284	1.627	0.380	0.185	0.782
Diagnosis of Unipolar Major depression	0.806	0.328	1.979	0.533	0.259	1.097
Diagnosis of cyclothymic disorder	0.795	0.564	0.956	0.564	0.254	0.782
Presence of psychotic symptoms during affective episodes, yes	0.800	0.471	1.361	1.087	0.670	1.762
Bis-11 Total Score ≥ 72	3.889 ****	2.298	6.579	2.668 ***	1.745	4.079
Poor free-interval functioning	1.720 *	1.053	2.808	1.698 *	1.112	2.591
Number of affective episodes	1.078 **	1.028	1.130	1.082 ***	1.032	1.134
Psychiatric hospitalizations, yes	4.450 ****	2.673	7.407	2.701 ***	1.714	4.256
Number of involuntary hospitalizations	0.778	0.549	1.102	0.768	0.544	1.083
Seasonality, yes	0.978	0.612	1.564	1.405	0.931	2.119
Cyclothymic temperament	1.083 *	0.505	2.322	1.026	0.528	1.992
Depressive temperament	1.480	0.703	3.116	1.672 *	0.886	3.156
Irritable temperament	1.188 *	0.467	3.025	2.152 **	0.964	4.803
Hyperthymic temperament	0.798 **	0.352	1.810	0.700 *	0.342	1.430
Anxious temperament	0.002	−0.234	0.289	0.002	−0.234	0.289

* *p* < 0.05; ** *p* < 0.01; *** *p* < 0.001; **** *p* < 0.0001.

**Table 3:** Linear regression analyses. Dependent variable: Number of lifetime suicide attempts.

	B	95% CI	
		Lower Bound	Upper Bound
Diagnosis of bipolar disorder	−0.109	−0.352	0.133
Diagnosis of Unipolar major depression	−0.035	−0.279	0.209
Diagnosis of cyclothymic disorder	−0.215	−0.365	0.854
Presence of psychotic symptoms during affective episodes, yes	−0.037	−0.202	0.128
Bis-11 Total Score ≥ 72	0.337 ***	0.204	0.470
Poor free-interval functioning	0.182 **	0.048	0.315
Number of affective episodes	0.020 **	0.007	0.034
Psychiatric hospitalizations, yes	0.495 ***	0.341	0.649
Number of involuntary hospitalizations	0.003	−0.114	0.120
Seasonality, yes	0.009	−0.131	0.148
Cyclothymic temperament	0.142 *	−0.169	0.353
Depressive temperament	0.012	−0.190	0.214
Irritable temperament	0.073	−0.352	0.206
Hyperthymic temperament	−0.141 **	−0.355	0.374
Anxious temperament	0.015	−0.124	0.189

* *p* < 0.05; ** *p* < 0.01; *** *p* < 0.001.

## Data Availability

The data presented in this study are available on request from the corresponding author.
